# Revolutionary ZVI-Entrapped Sol–Gel Silica Matrices: Efficient Catalytic Reduction of High-Concentration Halo-Organic Compounds—Addressing Bromoacetic Acid Contamination in Industrial Wastewaters

**DOI:** 10.3390/gels10110718

**Published:** 2024-11-07

**Authors:** Gifty Sara Rolly, Dan Meyerstein, Ariela Burg, Dror Shamir, Yael Albo

**Affiliations:** 1 Chemical Sciences Department and The Radical Research Center, Ariel University, Ariel 4070000, Israel; giftysrolly@gmail.com (G.S.R.); danm@ariel.ac.il (D.M.); 2 Chemistry Department, Ben-Gurion University, Beer-Sheva 8410501, Israel; 3 Chemical Engineering Department, Sami Shamoon College of Engineering, Beer-Sheva 84100, Israel; arielab@sce.ac.il; 4 Nuclear Research Center Negev, Beer-Sheva 84190, Israel; drorshamir@gmail.com; 5 Chemical Engineering Department and The Radical Research Center, Ariel University, Ariel 4070000, Israel

**Keywords:** de-halogenation, halo-organic compounds, sol–gel, wastewater, zero-valent iron

## Abstract

The de-halogenation of highly concentrated halo-organic compounds using Zero Valent Iron entrapped in silica matrices as a catalyst was investigated. This study aimed to evaluate the effectiveness of the Zero Valent Iron-entrapped organically modified silica matrices in transforming highly concentrated hazardous halogenated compounds into environmentally benign materials in the presence of BH_4_^−^. The Zero Valent Iron-entrapped silica gel matrices were synthesized using the sol–gel method. The de-halogenation products were analyzed using high-performance liquid chromatography. The results suggest that the Zero Valent Iron-entrapped silica matrices are effective catalysts in the de-halogenation reaction of halo-organics by BH_4_^−^ with 100% efficiency. The current work also highlights the complete de-bromination of harmful wastewater generated by the bromoacetic acid manufacturing industry using Zero Valent Iron-entrapped silica matrices. Therefore, Zero Valent Iron-entrapped silica matrices can be considered potential candidates for the catalytic removal of highly concentrated halo-organic compounds from contaminated water. This technology can play a crucial role in reducing the environmental impact of hazardous substances.

## 1. Introduction

Halogenated organic compounds are considered one of the major groups of environmental polluting chemicals that are used in industries and agriculture [1,2,3]. The massive release and extensive dissemination of these chemicals into the environment generates undesirable environmental contamination, leading to a deleterious impact on the environment and serious damage to human health and aquatic systems due to their toxic and carcinogenic effects [4]. Hence, this calls for the urgent development of advanced technologies with effective, inexpensive methods for the degradation of these compounds to resolve water contamination.

Chloro- and bromo-organic compounds are manufactured extensively for organic synthesis and flame-retardants [5]. Since they are xenobiotic organic compounds, they are fairly resistant to biodegradation and, hence, accumulate in the environment [4]. Several studies have been conducted in past years to develop efficient methods to eliminate these compounds from contaminated water. The most widely chosen method was reductive de-halogenation, which converts the halogenated compounds into less harmful substances [6,7]. Another method for the removal of halogenated compounds is biodegradation. While these compounds are resistant to biodegradation, some microorganisms have been found to be capable of breaking them down. Biodegradation can be achieved using bioreactors or by introducing appropriate microorganisms into the contaminated environment [8,9,10]. Advanced oxidation processes (AOPs) can also be used for the removal of halogenated compounds from contaminated water. AOPs use highly reactive species, such as hydroxyl radicals, to break down the compounds into less harmful substances [11,12,13,14,15,16].

A significant number of industries discharge highly concentrated halo-organic compounds into water sources, underscoring the need to develop effective methods for the degradation of concentrated halo-organic solutions. Generally, reductive de-halogenation is a widely used method for the elimination of chloro- and bromo-organic compounds from contaminated water [17,18]. In recent years, an efficient and cost-effective method using zero-valent metals for the reduction of chlorinated organic compounds at room temperature has been shown to be a promising method for treating contaminated underground water and industrial wastewater [19,20,21,22,23,24]. Among them, zero-valent iron (ZVI) technology has been extensively developed for industrial wastewater treatment, as iron is abundant, cost-effective, eco-friendly, and the remediation process is easily manipulated [25]. The study of the de-chlorination of compounds like chlorinated aliphatics [26,27] and chlorinated aromatics [24] manipulating ZVI has been very active since the innovative works of Gillham and O’Hannesin [26], as well as Matheson and Tratnyek in 1994 [27]. ZVI is commonly used in the de-halogenation of halo-organic compounds in both batch processes and by the injection of ZVI into contaminated underground water streams. However, this process can cause the release of iron ions into the water streams [28]. Also, these processes are slow in neutral and slightly alkaline conditions due to the formation of iron oxides/hydroxides on the surface of the zero-valent iron nanoparticles (ZVI-NPs). An alternative approach is stabilizing the ZVI as a heterogenous catalyst without the leaching of the metal ions.

Sol–gel silica entrapped nanoparticles are one of the most widely studied heterogeneous catalysts that have been applied in various catalytic processes due to their unique properties and high catalytic activity [29,30,31,32,33,34]. Adhikari et al. investigated the catalytic de-halogenation of halo-organic compounds using noble metal nanoparticles (gold and silver) entrapped in sol–gel matrices [28]. The study used sodium borohydride as the reducing agent for the de-halogenation of Br_3_CCO_2_^−^, Br_2_CHCO_2_^−^, BrCH_2_CO_2_^−^, CH_3_CHBrCO_2_^−^, CH_2_BrCH_2_CO_2_^−^, CH_2_BrCHBrCO_2_^−^, Cl_3_CCO_2_^−^, Cl_2_CHCO_2_^−^, and ClCH_2_CO_2_^−^, with the final concentration of all the halo-organic compounds set at 1.4 × 10^−3^ M. As noble metals are precious, their employment in industrial wastewater treatment is quite inconvenient. A recent study reported that ZVI entrapped in sol–gel matrices is an effective heterogeneous catalyst for reductive de-halogenation of halo-acetic acids [35] and chloro-acetamides [36] by BH_4_^−^. Neelam et al. used ZVI entrapped in sol–gel matrices to reduce 1.5 × 10^−3^ M concentrations of Br_3_CCO_2_H, BrCH_2_CO_2_H, Cl_3_CCO_2_H, and ClCH_2_CO_2_H; however, complete de-halogenation was not achieved. The study on the de-halogenation of chloro-acetamides (8.8 × 10^−3^ M) by Meistelman et al. demonstrated that ZVI entrapped in sol–gel matrices could completely reduce dichloroacetamide and monochloroacetamide to produce acetamide and acetic acid. The versatility of the sol–gel method for trapping ZVI in organically modified silica (ORMOSIL) enhances the catalyst’s performance. However, all the studies published so far have focused on the reduction of low concentrations of halo-organic compounds. Addressing high concentrations of halo-organic compounds is crucial for protecting both the environment and human health.

Given the critical importance of developing methods to degrade highly concentrated halo-organic compound wastes, our present work highlights the efficiency of zero-valent iron as a catalyst for converting concentrated, hazardous halogenated waste into environmentally friendly materials. Improper handling of industrial waste can have serious environmental and legal consequences. Hence, it is crucial for industrial plants to have well-defined waste management plans and to adhere to best practices to protect both the environment and public health. In the current work, a heterogeneous catalyst was designed, which consists of zero-valent iron nanoparticles encapsulated in an organically modified silica matrix (ZVI@ORMOSIL) to reduce highly concentrated halogenated substrates. The significance of the system is the mild reaction conditions and the high conversion values obtained. The key feature of this work is the employment of the catalyst in the removal of hazardous wastes in water from the bromoacetic acid (BAA) manufacturing industry.

## 2. Results and Discussion

### 2.1. Characterization of Matrices

**Figure 1:**
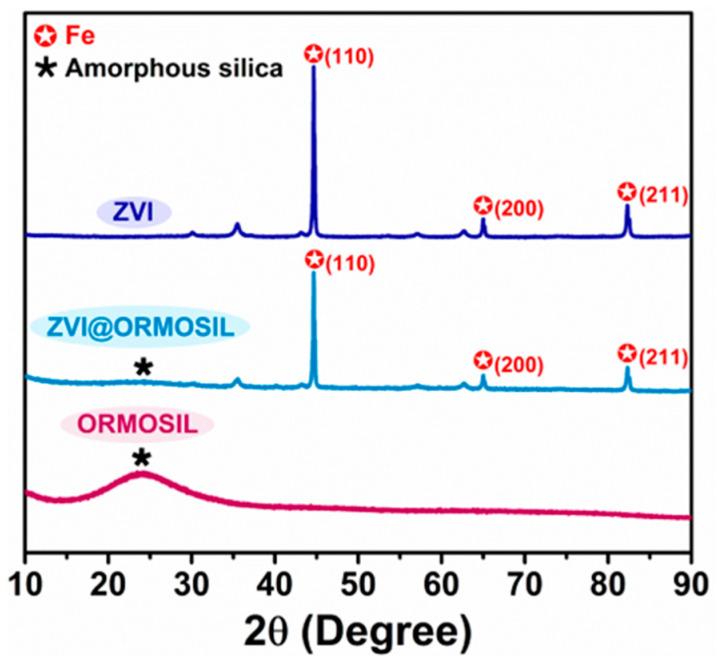
Powder XRD pattern of blank ORMOSIL, ZVI@ORMOSIL, and ZVI.

**Figure 2:**
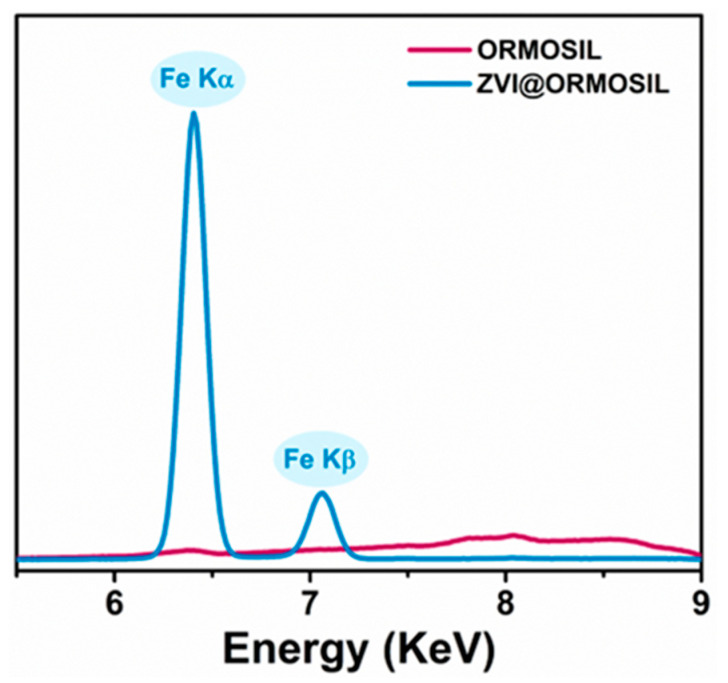
X-ray fluorescence analysis of blank ORMOSIL and ZVI@ORMOSIL.

**Figure 3:**
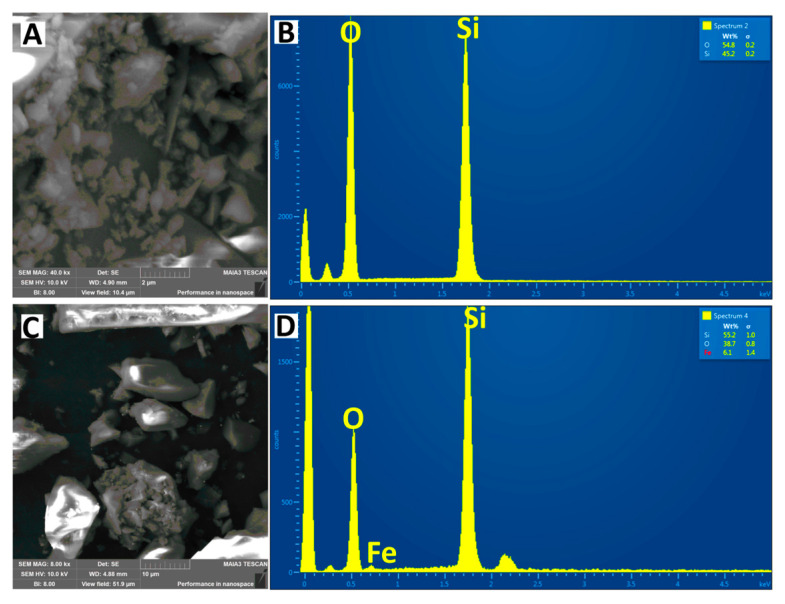
Scanning electron microscopy images and their corresponding Energy-dispersive X-ray spectroscopy (EDX) images of blank ORMOSIL (**A**,**B**) and ZVI @ORMOSIL (**C**,**D**).

The powder X-ray diffraction (XRD) patterns obtained for blank ORMOSIL, ZVI@ORMOSIL, and ZVI-NP powder are provided in Figure 1. The broad peak at 2θ = 22° observed in blank ORMOSIL and ZVI@ORMOSIL confirms the amorphous nature of the silica [37]. The observed peaks at 2θ of 44.63°, 64.94°, and 82.34° in ZVI@ORMOSIL correspond to (110), (200), and (211) reflection planes of iron (JCPDS: 006-0696) [38]. X-ray fluorescence (XRF) analysis was performed to ensure the presence of ZVI in the synthesized catalyst. Figure 2 shows the data obtained for blank ORMOSIL and ZVI@ORMOSIL. The XRF measured for ZVI@ORMOSIL exhibits peaks associated with Kα and Kβ lines of ZVI at 6.4 keV and 7.06 keV. The analysis reveals that the weight percentage of ZVI obtained was approximately 17%, which corresponds to the ZVI content present in the matrices. Scanning electron microscopy (SEM) and Energy-dispersive X-ray spectroscopy (EDX) measurements were performed to assess the morphology and the composition of the catalyst. Figure 3 depicts the SEM images and EDX spectra of blank ORMOSIL (A and B) and the ZVI@ORMOSIL catalyst (C and D). The SEM images of the blank and ZVI doped matrices show amorphous silica monoliths, which are characteristic of matrices prepared by the sol–gel synthesis route. A comparison of the two images indicates the amorphous nature of both matrices and that the morphology did not change significantly upon entrapment of the ZVI NPs. The prominent peaks observed in the EDX spectra for Si and O clearly originate from the matrices. The low weight percentage of Fe^0^ nanoparticles observed on the surface of the matrix indicates that most of the nanoparticles are entrapped in the pores of the silica.

**Figure 4:**
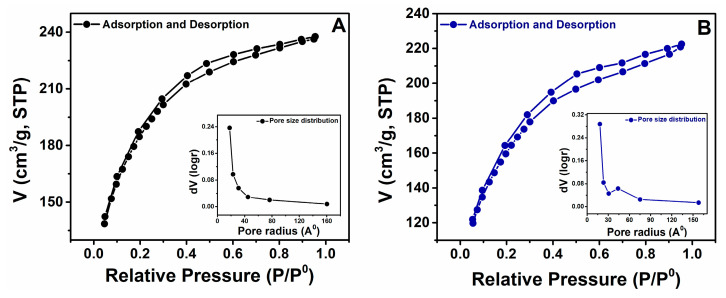
N_2_-adsorption–desorption isotherm of blank ORMOSIL (**A**) and ZVI@ORMOSIL (**B**). Inset: Pore size distribution.

To gain a better understanding of the pore systems in these materials, nitrogen adsorption–desorption isotherms and pore distributions were analyzed for both the ZVI@ORMOSIL catalyst and the blank ORMOSIL matrix. As shown in Figure 4, both materials exhibit a Type IV N_2_ adsorption–desorption isotherm, which is a characteristic feature of mesoporous materials (pores between 2 and 50 nm). The Brunauer–Emmett–Teller (BET) surface area and pore volume for the ZVI@ORMOSIL catalyst were 558 m^2^g^−1^ and 0.34 cm^3^g^−1^, respectively, while, for the blank ORMOSIL, they were 629.45 m^2^g^−1^ and 0.37 cm^3^g^−1^. The average Barrett–Joyner–Halenda (BJH) pore diameters for ZVI@ORMOSIL and blank ORMOSIL are 2.5 and 2.2 nm, respectively, which confirms their mesoporous nature. Upon entrapment of ZVI, slight changes in the shape of the hysteresis loop were observed (Figure 4B). The decrease in the BET surface area, the pore volume and pore diameters for ZVI@ORMOSIL compared to the blank ORMOSIL is due to the iron particles occupying space within the pores of the silica matrix. The higher density of ZVI reduces the overall accessible surface area available for gas adsorption as measured by BET analysis.

### 2.2. ZVI@ORMOSIL Catalyzed De-Halogenation of Halo-Organics Using NaBH_4_

**Figure 5:**
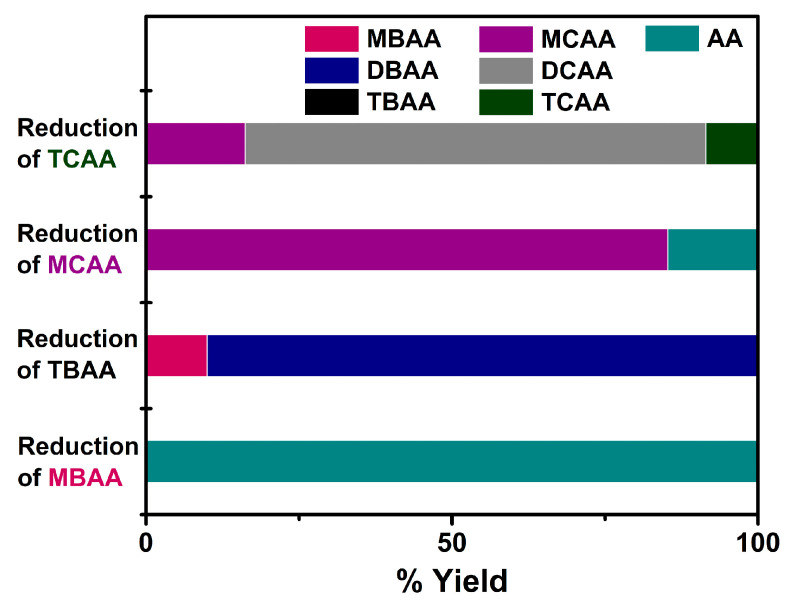
ZVI@ORMOSIL catalyzed the reduction of 0.4 M of MBAA, TBAA, MCAA, and TCAA at a 1:3 [Substrate]/[NaBH_4_] molar ratio. Total volume = 20.0 mL.

At the first stage, de-halogenation experiments were performed for mono-bromo-acetic acid (MBAA), tri-bromo-acetic acid (TBAA), mono-chloro-acetic acid (MCAA), and tri-chloro-acetic acid (TCAA) at a [Substrate]/[NaBH_4_] molar ratio of 1:3 in the presence of ZVI@ORMOSIL (Figure 5). A 100% reduction to acetic acid (AA) was obtained only in the case of MBAA. Product distributions of 90% DBAA and 10% MBAA for TBAA; 85% MCAA and 15% AA for MCAA; and 9% TCAA, 75% DCAA, and 16% MCAA for TCAA reduction were obtained.

**Figure 6:**
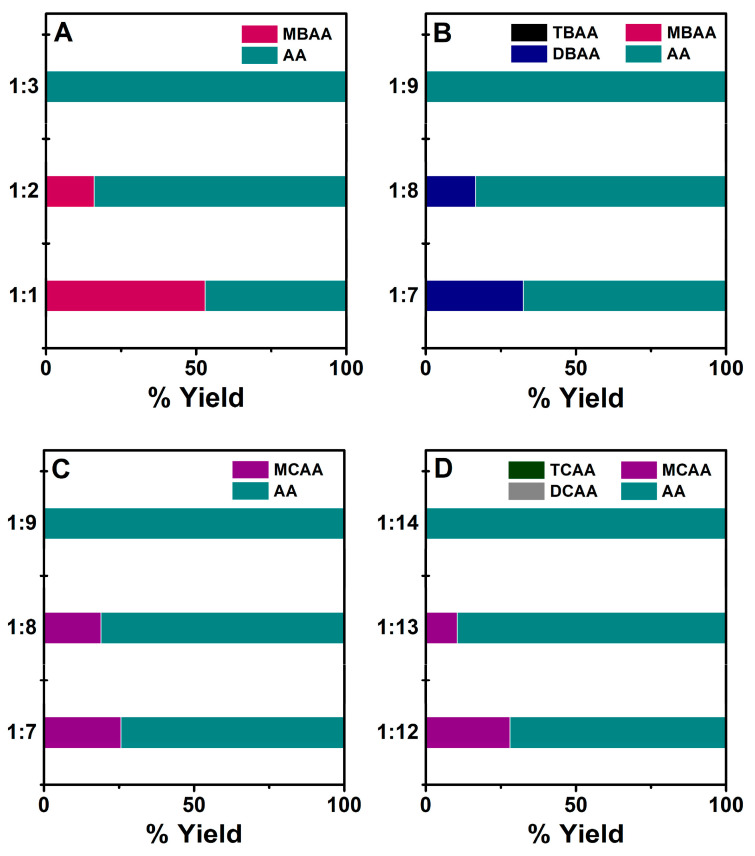
ZVI@ORMOSIL catalyzed the reduction of 0.4 M of (**A**) MBAA, (**B**) TBAA, (**C**) MCAA, and (**D**) TCAA at different [Substrate]/[NaBH_4_] molar ratios. Total volume = 20.0 mL.

To improve the de-halogenation percentage, the effect of increasing the relative excess of NaBH_4_ was examined. Therefore, the reduction was carried out using different [Substrate]/[NaBH_4_] ratios to determine the specific ratio at which 100% de-halogenation is achieved (Figure 6). The reduction of MBAA was tested using [Substrate]/[NaBH_4_] molar ratios of 1:1 and 1:2 to determine if complete dehalogenation could be achieved with a lower [NaBH_4_]. The products obtained were 47% AA for the 1:1 ratio and 84% AA for the 1:2 ratio, with the remaining fraction consisting of unreduced MBAA. Therefore, it became evident that a 1:3 [Substrate]/[NaBH_4_] molar ratio is necessary for the complete conversion of MBAA to AA (Figure 6A).

For the reduction of TBAA and MCAA, the [Substrate]/[NaBH_4_] molar ratios used were 1:7, 1:8, and 1:9. The yields obtained in the case of TBAA were 32% DBAA (di-bromo-acetic acid) and 68% AA for the 1:7 ratio, 16% DBAA and 84% AA for the 1:8 ratio, and 100% AA for the 1:9 ratio (Figure 6B). For MCAA, 75% and 81% AA were formed for the 1:7 and 1:8 ratios, respectively, with the remaining yield consisting of unreacted MCAA. At the 1:9 ratio, a 100% reduction to AA was achieved (Figure 6C). The dehalogenation of TCAA was performed with relatively higher [Substrate]/[NaBH_4_] molar ratios of 1:12, 1:13, and 1:14. The product distributions achieved were 28% MCAA and 72% AA for the 1:12 ratio, 10% MCAA and 90% AA for the 1:13 ratio, and 100% AA for the 1:14 ratio (Figure 6D).

**Figure 7:**
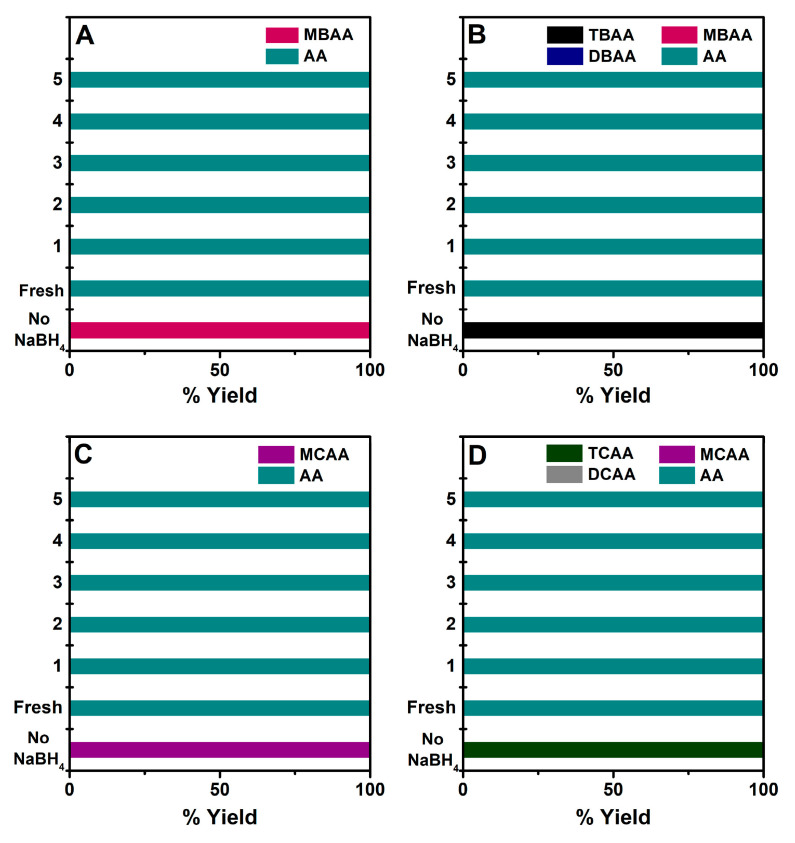
De-halogenation products obtained for 0.4 M of (**A**) MBAA, (**B**) TBAA, (**C**) MCAA, and (**D**) TCAA in the presence of ZVI@ORMOSIL. The reusability tests conducted for each reaction are represented by 1–5 in the graphs. [substrate]/[NaBH_4_] = (**A**) 1:3, (**B**) 1:9, (**C**) 1:9, and (**D**) 1:14. Total volume = 20 mL.

To evaluate the catalyst’s stability, reusability tests were performed for the reduction of MBAA, TBAA, MCAA, and TCAA. The matrix containing the catalyst was washed thoroughly with de-aerated water between each reaction cycle. This washing step was essential to remove all residual products from the previous reaction, ensuring that no by-products or unreacted species would interfere with the subsequent cycles. After washing, the matrix was dried before being reused in the next reaction. The use of de-aerated water was to prevent oxidation that could affect the catalyst’s surface. Figure 7 displays the complete de-halogenation for at least six reaction cycles and hence ensures the stability of the catalyst.

The de-bromination of MBAA and TBAA, conducted at a concentration of 0.4 M, achieved complete de-bromination at [Substrate]/[NaBH_4_] molar ratios of 1:3 and 1:9, respectively, with both substrates being efficiently reduced to acetic acid within an hour, indicating that ZVI@ORMOSIL is an efficient catalyst. The degradation pathway of TBAA involves sequential transformations leading to the formation of DBAA, then to MBAA, and finally to acetic acid. Like TBAA, the degradation of TCAA also follows a sequential transformation pathway. It proceeds from TCAA to DCAA (di-chloro-acetic acid) and further to MCAA, ultimately resulting in the formation of acetic acid (AA). The reactions are much slower than those of TBAA and MBAA de-halogenation due to the stronger C-Cl bond. [Substrate]/[NaBH_4_] molar ratios of 1:9 and 1:14 are required for MCAA and TCAA for complete conversion to acetic acid. It is proposed that the mechanisms of the de-halogenations proceed via the following reactions (Equations (1)–(7)), in analogy to a previously suggested mechanism [35].

ZVI-NP + (n/4)BH_4_^−^ + (3/4)nH_2_O → (ZVI-NP)-H_n_^n−^ + (n/4)B(OH)_3_ + (3n/4)H^+^ (1)

(ZVI-NP)-H_n_^n−^ + mH_2_O → (ZVI-NP)-H_n+m_^(n−m)−^ + mOH^−^ (2)

(ZVI-NP)-H_n+m_^(n−m)−^ + X_k_H_3−k_CCO_2_^−^ → (ZVI-NP)-H_n+m−1_^(n−m)−^ + X_k−1_H_3−k_CCO_2_^−^ + X^−^ + H^+^ (3)

(X = Cl or Br; k = 3 or 2 or 1) (ZVI-NP)-H_n+m_^(n−m)−^ + X_k_H_3−k_CCO_2_^−^ → (ZVI-NP)-H_n+m_^(n−m−1)−^ + X_k−1_H_3-k_CCO_2_^−^ + X^−^ (4)

{(ZVI-NP)-H_n+m−1_}^(n−m)−^ + X_k−1_H_3−k_CCO_2_^−^ → {(ZVI-NP)-H_n+m−1_}-CX_k−1_H_3−k_CO_2_^(n−m+1)−^ (5)

{(ZVI-NP)-H_n+m−1_}-CX_k−1_H_3-k_CO_2_^(n−m+1)−^ → {(ZVI-NP)-H_n+m−2_}^(n−m)−^ + CX_k−1_H_4−k_CO_2_^−^ (6)

{(ZVI-NP)-H_n+m−1_}-CX_k−1_H_3-k_CO_2_^(n−m+1)−^ + H_2_O → {(ZVI-NP)-H_n+m−1_}^(n−m−1)−^ + CX_k−1_H_4−k_CO_2_^−^ + OH^−^ (7)

**Scheme 1:**
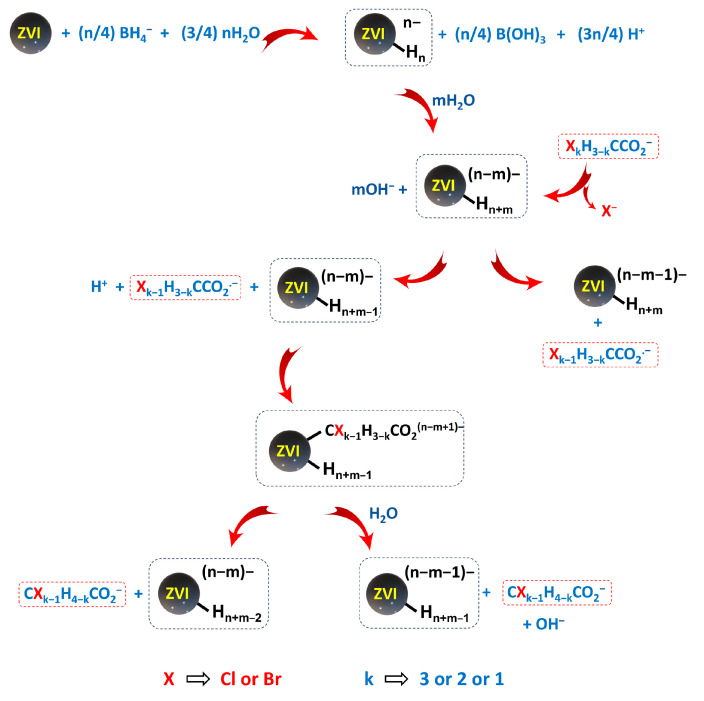
Schematic representation of the reaction mechanism (Equations (1)–(7)).

Reaction (1) is commonly accepted for the M^0^-NPs catalyzed hydrolysis of BH_4_^−^ [28]. However, recent DFT studies on BH_3_^−^ hydrolysis on Ag^0^-NPs [39,40] suggest a more complex hydrolysis mechanism. The detailed mechanism for the ZVI catalyzed hydrolysis of BH_4_^−^ is not known. This might affect Reactions (1)–(4). The schematic representation of the mechanism is illustrated in Scheme 1.

**Figure 8:**
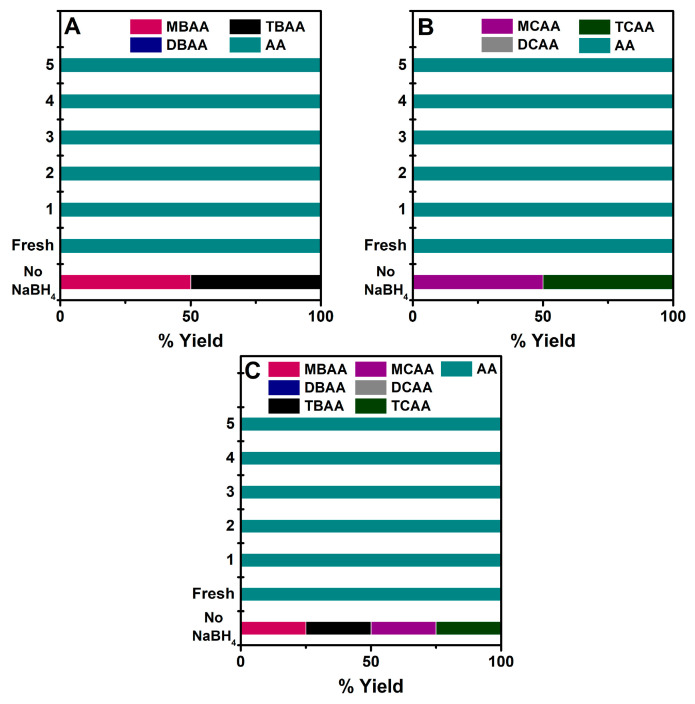
De-halogenation products obtained for the first portion of substrate combinations 0.4 M (**A**) MBAA+TBAA, (**B**) MCAA+TCAA, and (**C**) MCAA+TCAA+MBAA+TCAA in the presence of ZVI@ORMOSIL. The reusability tests conducted for each reaction are represented by 1–5 in the graphs. [substrate]/[NaBH_4_] = (**A**) 1:6, (**B**) 1:11, and (**C**) 1:15. Total volume = 20 mL.

As waste streams frequently consist of multiple components, we conducted additional assessments of the catalyst’s efficacy in reducing substrate mixtures like MBAA+TBAA, MCAA+TCAA, and MCAA+TCAA+ MBAA+TBAA using NaBH_4_ as the reducing agent in a total reaction volume of 20 mL (Figure 8). The ZVI@ORMOSIL catalyzed de-halogenation reaction of both individual substrates, and their mixtures exhibited complete de-halogenation, resulting in the production of acetic acid (AA) as the final product. The reusability of the catalyst was also evaluated, and it demonstrated stability for at least six reaction cycles, achieving complete dehalogenation.

The first stage reactions of substrate mixtures were conducted at a total substrate concentration of 0.4 M (0.2 M of each substrate) in the case of MBAA+TBAA and MCAA+TCAA and 0.4 M (0.1 M of each substrate) for MCAA+TCAA+MBAA+TBAA. The MBAA+TBAA mixture was completely reduced within 1 h at a substrate to BH_4_^−^ molar ratio of 1:6. The reduction of MCAA+TCAA mixtures to complete acetic acid was achieved at a substrate to BH_4_^−^ molar ratio of 1:11. However, the de-chlorination of the MCAA+TCAA+MBAA+TBAA mixture required a higher substrate to BH_4_^−^ molar ratio of 1:15. The de-chlorination reaction required approximately 16 h to reach completion.

**Figure 9:**
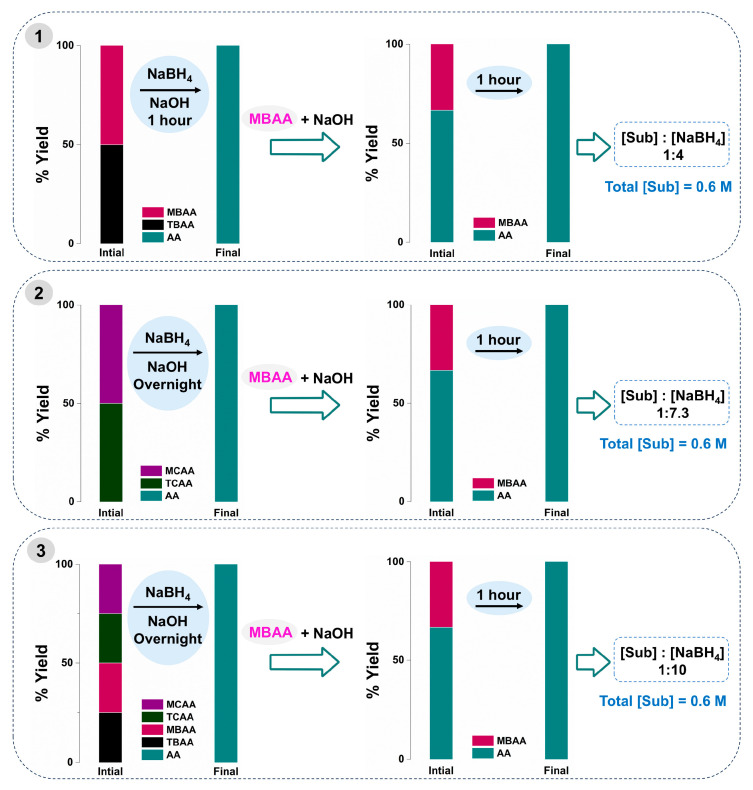
De-halogenation products of the first and second stages of substrate mixture reactions.

**Scheme 2:**
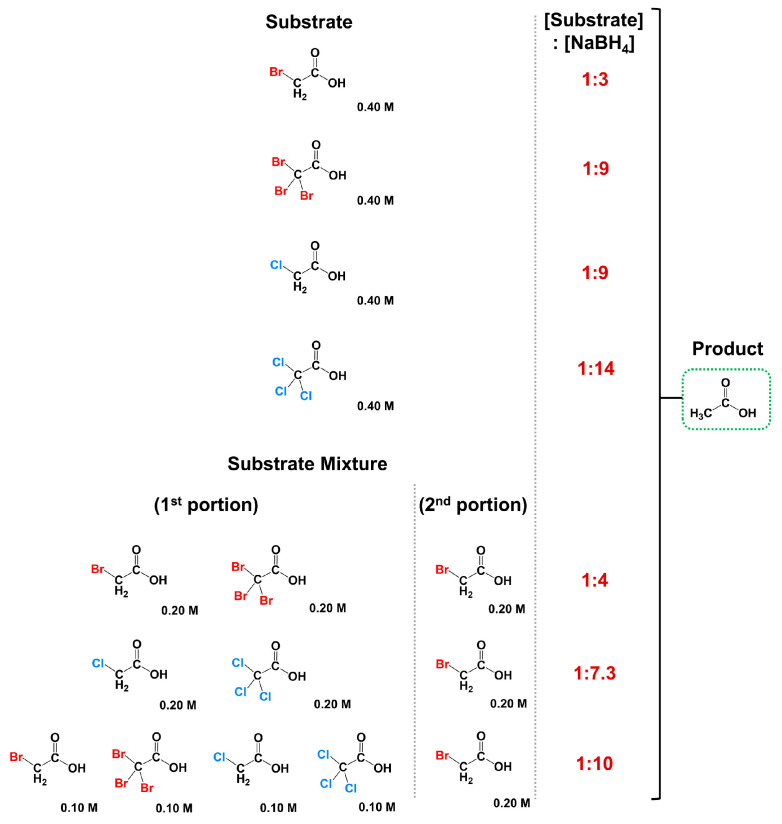
[Substrate]/[NaBH_4_] molar ratio required to reduce substrate and their mixtures to yield a complete reduction to acetic acid (AA).

To create an environmentally friendly process, it was decided to explore the possibility of decreasing the volume of solvents and reducing agents utilized in the procedure. As a second stage of the reaction, solid MBAA (0.2 M) was subsequently introduced following the completion of de-halogenation reactions of all three mixtures. The results obtained are provided in Figure 9. The complete de-halogenation was obtained in 1 h for all the above-mentioned reactions without introducing additional NaBH_4_ into the mixture. The total substrate to BH_4_^−^ molar ratios required to reduce each substrate mixture (first and second stages) are depicted in Scheme 2.

### 2.3. De-Halogenation of Industrial Waste Containing Bromoacetic Acids

The de-halogenation of industrial waste containing bromoacetic acids was performed using a total concentration of 0.80 M waste solution containing MBAA (70%) and DBAA (30%) mixture. From the high-performance liquid chromatography (HPLC) analysis, the concentrations of bromoacetic acids present in the bulk solution of the industrial waste were [MBAA] = 7.52 M and [DBAA] = 3.2 M. The de-bromination reaction was carried out using a [substrate] to [NaBH_4_] ratio of 1:4. The reaction conditions are provided in Table 1.

**Figure 10:**
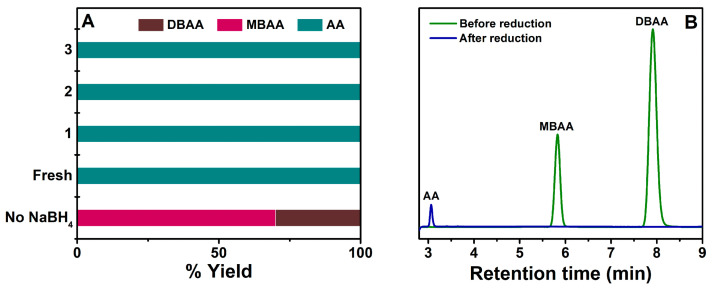
(**A**) The de-halogenation of industrial waste containing a total concentration of 0.8 M of MBAA (70%) and DBAA (30%) mixture. The reusability tests conducted for each reaction are represented by 1–3 and (**B**) their corresponding HPLC chromatogram representing the peaks before and after reduction. [substrate]/[NaBH_4_] = 1:4. Total volume = 100 mL.

Complete de-bromination was achieved at this concentration of bromoacetic acids mixture. Reusability tests were also conducted to ensure the stability of the catalyst (Figure 10A). The chromatogram obtained is provided in Figure 10B.

## 3. Conclusions

The catalytic degradation of halo-organic compounds using a ZVI-entrapped silica sol-gel matrix displayed a complete de-halogenation of all the substrates and substrate mixtures into acetic acid (AA) as the final product. It should be pointed out that the presence of BH_4_^−^ inhibits the formation of the oxide/hydroxide protecting layer of the ZVI. The complete degradation of highly concentrated halo-organic compounds is a significant advancement in waste remediation and environmental protection. The transformation of halo-organic compounds into acetic acid (AA) as the final product is not only an environmentally friendly process but also potentially economically valuable. Furthermore, a 100% de-bromination was attained in the reduction of a waste solution obtained from the bromoacetic acid (BAA) production industry. It indicates that the ZVI-entrapped silica matrix method can be applied directly to address specific industrial waste streams, making it a practical and potentially lucrative solution for industrial waste management.

## 4. Materials and Methods

### 4.1. Materials

All commercial chemicals were of A.R. grade. Iron nano-powder (50 nm, 99.9%) was purchased from Glentham-Life Sciences (Corsham, UK). Mono-bromo-acetic acid (MBAA) (99%), tribromo-acetic acid (TBAA) (97%), tetraethyl ortho-silicate (99%) (TEOS), and tri-methoxy-methyl-silane (98%) (MTMOS) were purchased from Sigma-Aldrich, and Mono-chloro-acetic acid (MCAA) (99%) and tri-chloro-acetic acid (TCAA) (99%) from Alfa Aesar. Sodium borohydride (NaBH_4_) (98%) was purchased from Thermo Scientific. NH_3_ solution (30%) was purchased from Carlo Erba, ethanol and hydrochloric acid (37%) from Bio-Lab Ltd., Jerusalem, Israel. An industrial waste solution containing concentrated bromoacetic acid was obtained from one of the industrial plants. All chemicals were used without further purification. Water purified with a Milli-Q system with a resistivity > 15 MΩ·cm^−1^ was used throughout the experiments.

### 4.2. Instrumentation

Powder X-ray diffraction (PXRD) measurements of the matrices were carried out using a Rigaku SmartLab SE X-ray diffractometer (Akishima, Japan) using Cu-Kα (1.54 Å) radiation operated at 40 kV and 30 mA. X-ray fluorescence (XRF) was measured using a Portable X-ray Fluorescence (pXRF), Niton XL3t GOLD + analyzer instrument (Thermo Fisher Scientific, Waltham, MA, USA). SEM analysis was performed using 3FE-Tescan ultra-high-resolution MAIA microscope with an AZTEC microanalysis EDX detector, Oxford Instruments, Concord, MA, USA. HPLC analysis was performed using a Dionex Ultimate 3000 equipped with a diode array detector (Thermo Scientific, Germering, Germany) .

### 4.3. Synthesis of ZVI@ORMOSIL Catalyst

The catalyst was synthesized through a two-step acid/base sol–gel process [35]. To a 100 mL beaker containing 8.8 mL of ethanol, 6.0 mL TEOS was added with stirring, followed by the addition of 1.6 mL MTMOS. The stirring continued for 15 min from the time of the addition of MTMOS. Later, 2.7 mL HCl (0.277 M) was added dropwise and stirred for another 15 min. Thereafter, 2.0 mL NH_3_ solution (2.5%, 0.513 M) was added dropwise, followed by the addition of ZVI NP suspension (1.0 M in ethanol) to give 30% mol of ZVI@ORMOSIL. The solution was stirred for several minutes until the gelation occurred. After aging and drying the solid gel for 14 days, the resulting solid matrix was washed multiple times with de-aerated water. The matrix was subsequently dried, crushed into a powder using a mortar and pestle, and used as a catalyst for the experiments.

### 4.4. De-Halogenation Reactions Using the ZVI@ORMOSIL Catalyst

The de-halogenation of halo-organic compounds was performed using a 30% mole ZVI@ORMOSIL catalyst. In a typical catalytic reaction, solid substrates and their mixtures were accurately weighed and transferred into a 100 mL reaction bottle containing ca. 0.25 g of ZVI@ORMOSIL catalyst. Subsequently, 15 mL of water was added, followed by the addition of 5.0 mL NaOH solution to the mixture. The substrate and substrate mixture concentrations were 0.4 M for each batch process. The NaOH solution was introduced before each reaction to avoid excess H_2_ evolution during the addition of NaBH_4_ into the acidic substrate solutions. The concentration of total substrate and NaOH was kept equal in the final solution. The solution was then stirred for several minutes. The de-halogenation reaction was initiated by adding the necessary amount of solid NaBH_4_ in portions as the reducing agent. The suspensions containing MBAA and TBAA were stirred for 1 h, while those containing MCAA and TCAA were stirred overnight.

**Scheme 3:**
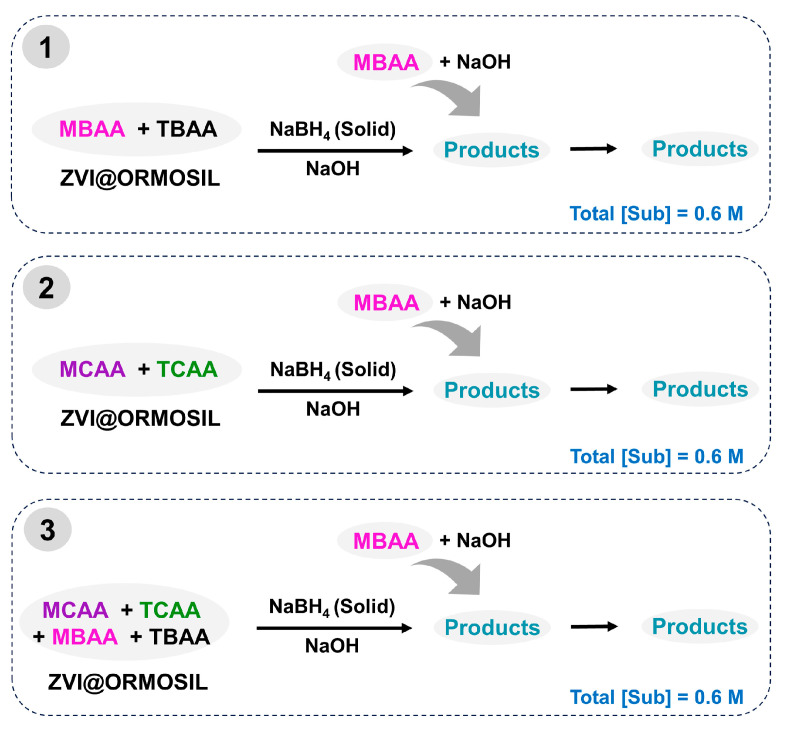
Presentation of substrate combination reaction procedure. (1) MBAA+TBAA (2) MCAA+TCAA and (3) MCAA+TCAA+MBAA+TBAA.

Furthermore, substrate combinations were subjected to de-halogenation. The first stage of the reaction was the reduction of the mixtures: MBAA+TBAA, MCAA+TCAA, and MCAA+TCAA+MBAA+TBAA. A second stage reaction was performed after the completion of de-halogenation reactions within the mixtures. Solid MBAA (0.2 M) was subsequently introduced into each of the reaction mixtures. The addition process is illustrated in Scheme 3.

Product analysis was carried out using HPLC after the completion of the reaction. The solutions were diluted and adjusted to pH 2.0 using H_3_PO_4_. All the solutions were filtered through a 0.22 µm PES filter membrane before analysis. HPLC analysis was carried out using a Dionex Ultimate 3000 equipped with a diode array detector (Thermo Scientific), Hypersil GOLD (C18, dim. (mm)—150 × 4.6, particle size—3 μm) column. The substrates were eluted using 0.2% H_3_PO_4_/CH_3_CN = 98:2, UV detection wavelength of 200 nm, temperature 25 °C, injection volume 30 μL, and a flow rate of 1.0 mL/min.

### 4.5. Reduction of Bromoacetic Acids in Industrial Wastewater

Waste solutions obtained from industry after the bromoacetic acid (BAA) production process were used in this study. A 100 mL sample of the required concentration of the bromoacetic acids waste containing an equal concentration of NaOH was mixed with 1.25 g of ZVI@ORMOSIL in a 500 mL beaker. Thereafter, the de-bromination was carried out by adding four portions of the required amount of solid NaBH_4_ as the reducing agent.

## Figures and Tables

**Table 1:** Conditions of complete debromination of BAA waste solution in the presence ZVI@ORMOSIL. Total volume = 100 mL.

[Substrates] (M)	[MBAA] ppm	[DBAA] ppm	[Substrate]/ [NaBH_4_]	Product
[MBAA]+ [DBAA] = 0.80 M	77,812	52,281	1:4	Acetic acid (AA)

## Data Availability

All data analyzed during this study are included in the article. Any further inquiries can be directed to the corresponding authors.
